# The Effect of Insulin Resistance on Ovulation Induction With Clomiphene Citrate in Non-polycystic Ovary Syndrome (PCOS) Women

**DOI:** 10.7759/cureus.27433

**Published:** 2022-07-29

**Authors:** Ruchi Karnatak, Anjoo Agarwal, Mona Asnani, Renu Singh

**Affiliations:** 1 Obstetrics and Gynaecology, King George's Medical University (KGMU), Lucknow, IND

**Keywords:** prospective cohort study, insulin resistance, ovulation induction, clomiphene citrate, anovulatory infertility

## Abstract

Objective

To study the status of insulin resistance (IR) in non-polycystic ovary syndrome (PCOS) infertile women and to assess its effects on response to ovulation induction (OI) with clomiphene citrate (CC).

Methods and materials

A prospective cohort study was conducted at the Department of Obstetrics and Gynecology, King George Medical University, Lucknow, India, over a one-year period. One hundred two women who underwent treatment for infertility were enrolled and evaluated for insulin resistance. Insulin resistance was assessed using fasting serum insulin levels and the Homeostatic Model Assessment of Insulin Resistance (HOMA-IR) index. All participants were given ovulation induction with clomiphene citrate and were then followed up for the occurrence of ovulation and conception.

Results

Insulin resistance was found in 20.5% of infertile subjects and 95.4% of anovulatory subjects. Of the subjects with insulin resistance, 80.5% showed monofollicular development. The pregnancy rate was 5.8%, but there was no conception among subjects with insulin resistance.

Conclusions

Insulin resistance was found to be present in 20.5% of infertile women. Women with insulin resistance are more likely to have monofollicular development on ovulation induction and are less likely to conceive as compared with women without insulin resistance (OR = 0.2079).

## Introduction

Infertility is a common condition that has psychological, economic, and medical implications. It has been defined as an inability to conceive after one year of regular, unprotected sexual intercourse [1]. Hyperinsulinemia may contribute to infertility. Insulin has an important role in ovarian steroidogenesis and hence in ovulation [2]. Hyperinsulinemia affects granulosa cells and theca cells. Due to hyperinsulinemia, luteinizing hormone induces early response on granulosa cells of small follicles and causes premature differentiation of theca cells. As a result, hyperinsulinemia causes anovulation. Insulin sensitizing agents are useful for the improvement of anovulation due to hyperinsulinemia.

In addition to ovulatory disturbances, hyperinsulinemia may adversely affect endometrial functions and evoke implantation disturbance. Treatment with an insulin-sensitizing agent (metformin) improves the endometrial environment, improves the level of glycodelin, insulin-like growth factor binding protein 1, and blood flow in spiral arteries in the peri-implantation period. This hypothesis is important to the Indian scenario, as India is known as "the diabetes capital of the world" and every fifth diabetic in the world is an Indian [3]. PCOS is often associated with profound insulin resistance as well as defects of insulin secretion. However, insulin resistance independent of PCOS may be a culprit of these ovulatory disturbances in infertility. Though there are many studies of insulin resistance in PCOS women there are no studies in non-polycystic ovary syndrome (PCOS) infertile women to know this independent effect of insulin resistance on ovulation.

Our study aimed to first ascertain the status of insulin resistance in non-PCOS infertile women and second, to study the effect of insulin resistance in subjects’ response to ovulation induction with clomiphene citrate.

## Materials and methods

A prospective cohort study was conducted over a period of one year (from September 2018 to August 2019). Ethical approval was obtained from the Institutional Ethical Committee, King George Medical University, Lucknow (IRB ECR/262/Inst/UP/2013/RR-16).

Subjects were enrolled from women who attended the infertility clinic at the Department of Obstetrics and Gynecology, KGMU, Lucknow. Each provided written, informed consent to participate in the study. Women undergoing treatment for infertility were included, excluding subjects who had poor ovarian reserve (serum anti-Mullerian hormone<1.1 and antral follicle count<7), untreated hypothyroidism or hyperprolactinemia, diabetes, or bilateral tubal block. Women meeting Rotterdam’s criteria for the diagnosis of PCOS were also excluded [4]. The minimum sample size was 100, calculated using the formula: n=z2× p × (1-p)/d2, requiring a p-value of 0.5 [5-6], so 102 women were recruited.

Subjects were stratified on the basis of body mass index (BMI) using Indian criteria [7]. Women with BMI ≥23 kg/m^2^ were labeled as overweight; those women with BMI ≥25 kg/m^2^ were labeled obese. Subjects were also categorized on the basis of the waist-to-hip ratio [7]. A full evaluation for the cause of infertility was performed. All subjects underwent the glucose tolerance test. Their fasting insulin levels were assessed using chemiluminescent immunoassay [8].

All subjects received ovulation induction with clomiphene citrate at a 100 mg dose from Day 2 to Day 6; follicular growth was monitored. When the dominant follicle reached preovulatory size (18-21 mm), an ovulation trigger was administered (Inj human chorionic gonadotropin (HCG) 10000 IU). Single intrauterine insemination was performed 36-48 hours following the administration of the ovulation trigger. All subjects were followed up for conception. Insulin resistance was calculated using the Homeostatic Model Assessment of Insulin Resistance (HOMA-IR) formula:

HOMA-IR = fasting serum glucose (mg/dl) ×fasting insulin (µlU/ml) / 405

HOMA-IR value ≥2 was taken to identify insulin resistance [9]. Outcome measures included the presence of insulin resistance in non-PCOS infertile women, the occurrence of ovulation, and the occurrence of conception.

Statistical analysis was performed using SPSS Version 21.0 software (IBM Corp., Armonk, NY).

## Results

A total of 102 women were enrolled with maximum subjects in the age group of 30-34 years (n= 44; 43.1%); the youngest subject was 22 years old and the oldest was 41 years old. The majority of subjects (n=82; 80.3%) were nulligravida (i.e. they had never previously conceived). A majority of subjects (n=66; 64.7%) were of the upper-middle class social scale, according to the modified Kuppuswamy socioeconomic scale. The majority of subjects (n=74; 72.5%) had a mean duration of infertility of one to four years. All subjects had a regular menstrual cycle with average blood flow. None had a family history of diabetes, hypertension, premature menopause, or any hormone-dependent malignancy.

There was a 20.5% prevalence of insulin resistance (based on HOMA-IR with a cut-off value of ≥2; Table 1). None of the subjects had overt diabetes.

**Table 1:** Distribution of HOMA-IR values in the study population Mean = 1.49 ± 0.95 HOMA-IR: Homeostatic Model Assessment of Insulin Resistance

Serial number	HOMA-IR	Number of women	Percentage (%)
1	<1	25	24.5
2	1–1.9	56	54.9
3	2–2.9	18	17.6
4	≥ 3	3	2.9
	Total	102	100

Of the subjects, 45% had normal BMI, 19.6% were overweight, and 35.2% were obese. Two point one percent (2.1%) of normal BMI women had HOMA-IR≥2, 2% of overweight women had HOMA-IR ≥2, and 18% of obese women had HOMA-IR ≥2. BMI and waist-to-hip ratio were found to be significantly correlated to insulin resistance on the basis of HOMA-IR ≥2 (p<0.001 and p<0.001, respectively; Tables 2-3).

**Table 2:** Correlation between BMI and HOMA-IR chi-square=30; p<0.001 HOMA-IR: Homeostatic Model Assessment of Insulin Resistance; BMI: body mass index

Serial number	BMI (kg/m^2^)	Total number of women	Number of women HOMA-IR ≥2	Percentage (%)	Relative risk of insulin resistance	95%confidence Interval	P-value
1	<18.5	0	0	0			
2	18.5–22.9	46	1	2.1			
3	23–24.9	20	2	10	4.6	0.4-47.8	0.20
4	≥25	36	18	50	23	3.2-164.2	<0.0018
	Total	102	21				

**Table 3:** Correlation of the waist-hip ratio with HOMA-IR Chi-square 32.1, p<0.001 HOMA-IR: Homeostatic Model Assessment of Insulin Resistance

Serial number	Waist: hip ratio	Number of women	Number of women with HOMA-IR ≥2	Percentage (%)	Relative risk of insulin resistance	95% confidence interval	P-value
1	<0.80	42	1	2.3			
2	0.80–0.84	22	1	4.5	1.9	0.12–29.0	0.6
3	≥0.85	38	19	50	21	2.95–149.4	0.0024
	Total	102	21				

All subjects developed at least one follicle of ≥18 mm; 55 of 102 women had monofollicular development. The number of dominant follicles significantly correlated with insulin resistance (based on HOMA-IR ≥2); insulin resistance decreased the probability of multifollicular development (p =0.005). Of the subjects who developed two dominant follicles, 12.7% (five of 41) conceived. Of those who developed three follicles, 16.3% (one of six) conceived. No conception was observed in subjects who had monofollicular development. The conception rate was highest in women who developed three follicles (Tables 4-5).

**Table 4:** Distribution of women based on conception

S. No.	Conception	Number of women N=102
1	No	96
2	Yes	6

**Table 5:** Distribution of conceived women based on number of developing follicles

Number of women conceived out of all women (n=102)	Number of women conceived out of women who developed 1 follicle (n=55)	Number of women conceived out of women who developed 2 follicles (n=41)	Number of women conceived out of women who developed 3 follicles (n=6)
6	0 (0%)	5 (12.2%)	1 (16.6%)

Ovulation trigger was administered to all subjects using an HCG 10,000 IU intramuscular injection when the dominant follicle reached preovulatory size (18-21 mm). They were followed up for dominant follicular rupture. It was found that there was no correlation between follicular rupture and insulin resistance.

A total of 21 women had insulin resistance (HOMA IR≥2). Eighty point ninety-five percent (80.95%) of subjects with insulin resistance (17 of 21) had monofollicular development; only 19.05% (4 out of 21) had multifollicular development. The associated p-value of 0.005 strongly indicates that insulin resistance is significantly negatively correlated with follicular development (Table 6).

**Table 6:** Correlation of number of dominant follicles ratio with HOMA-IR ≥2 (n=102) chi-square=7.77, p-value=0.005 HOMA-IR: Homeostatic Model Assessment of Insulin Resistance

S no.	No. of dominant follicles	No. of total women	No. of women with HOMA IR≥2	Percentage of women with HOMA IR>2 out of total women
1	1	55	17	30.9
2	2	41	3	7.3
3	3	6	1	16.6
	Total	102	21	

Out of 21 women with insulin resistance( HOMA IR≥2), 18 women (85.7%) had S. AMH≥4 (p<.001). It shows that insulin resistance is significantly correlated with anti-Müllerian hormone (AMH) levels (Table 7).

**Table 7:** Correlation of S. AMH with HOMA-IR ≥2 (n=102) Chi square=19.5, p value=<0.001 HOMA-IR: Homeostatic Model Assessment of Insulin Resistance; S. AMH: serum anti-Müllerian hormone

S no.	S AMH (ng/ml)	No. of women	HOMA-IR≥2		%	Relative risk
1	<1.0	0	0		0	
2	1.0-3.9	58	3		5.1	
3	≥4	44	18		40.9	7.9 (95% CI 2.4-25.1 p=0.0005)
	Total	102	21			

## Discussion

Our study found insulin resistance to be prevalent in 20.5% of cases of non-PCOS infertile women (based on HOMA-IR). A previous study by Al-Jefout et al. found insulin resistance in 133 women out of 159 with PCOS (83.6%) and in 25 women out of 54 without PCOS (46.3%) (p<0.001) [10].

In our study, obesity was correlated with anovulation. It was found that women with obesity (BMI ≥25 kg/m^2^ ) had significantly higher anovulation as compared to women with normal BMI (chi-square 28.4, p-value<0.00001). This was similar to a study by Grodstein F, Goldman, and Cramer [11] in which obese women (body mass index ≥27 kg/m^2^) had a relative risk of ovulatory infertility of 3.1 (95% confidence interval (CI) = 2.2-4.4), compared with women of lower body weight (body mass index 20-24.9 kg/m^2^). They concluded that the risk of ovulatory infertility is highest in obese women.

The treatment of infertility with clomiphene citrate (CC), followed by intrauterine insemination, is a useful infertility treatment when offered to an appropriate candidate. The pregnancy rate in our study was 5.8%, which is comparable to the rates reported in studies by Huang et al. and Soysaland Ozmen, in which pregnancy rates were 4.6% and 8.3%, respectively [12-13]. We observed no conception in subjects with insulin resistance (HOMA-IR ≥2).

Insulin resistance in this study was found to be inversely related to the number of developing follicles. There was no conception observed in women with monofollicular development while 12.7% of women with multifollicular development conceived. Similar results were reported by Vargas-Tominaga L et al., who found that having two or more follicles was the factor most associated with clinical pregnancy rates (11.6% with ≥2 follicles; 4.4% with ˂ 2 follicles; OR:2.283) [14].

In a study by Park SJ on the ovulatory status and follicular response to predict the success of clomiphene citrate-intrauterine insemination, it was found that in anovulatory women, the clinical pregnancy rates (CPR) and live birth rates (LBR) were 15.7% and 13.6%, respectively [15]. The study concluded that treatment with CC-IUI is more successful in anovulatory women than in ovulatory women. The multifollicular response in both ovulatory and anovulatory women increases pregnancy rates. In our study, 12.7% of the women who had development of ≥2 dominant follicles conceived, and no conception was seen in women with mono-follicular development.

A retrospective cohort study was done by Kort JD to evaluate the impact of meaningful weight loss on fertility outcomes in an overweight population with infertility [16]. In this study, patients were given a “meaningful” weight loss goal of 10 kg.

Our study evaluated one cycle of ovulation induction with clomiphene citrate. It is limited by its small sample size. For validation, it should be repeated in a larger population with more cycles of ovulation induction so as to validate and further define the importance of insulin resistance in the workup of infertile women.

## Conclusions

Insulin resistance was present in 20.5% of non-PCOS infertile women. Insulin resistance was found to be significantly correlated with BMI (p <0.001) and the waist-to-hip ratio (p<0.001). The rate of conception among our subjects was 5.8%. Most subjects with insulin resistance (80.5%) showed monofollicular development. There were no conceptions among the women with insulin resistance. Thus, insulin resistance can be considered a significant factor contributing to anovulation. Our study evaluated one cycle of ovulation induction with clomiphene citrate. The limitation of this study is that only one cycle of ovulation induction was studied and the sample size was also not very large. This kind of study should be repeated in a larger population with more cycles of ovulation induction so as to validate and further define the importance of insulin resistance in the workup of non-PCOS infertile women.
